# Tracking footprints of plastid evolution

**DOI:** 10.1007/s00709-020-01526-9

**Published:** 2020-06-22

**Authors:** Peter Nick

**Affiliations:** Botanical Institute, Karlsruher Institut für Technologie, Karlsruhe, Germany

Not rarely, we fail to understand a phenomenon alone from its current state; instead, we need to know how it came into being. The presence always encompasses the footprints of the past. This is prominently true for the evolution of cells. The way how organelles are organised, why they contain their own genomes and how they import most of the protein components by a regulated, complex and energy-consuming machinery is hard to derive from the current set-up of a cell. It is immediately understood, however, if described as remnants of an endosymbiotic event (Sagan 1967). To read a process from the footprints it left behind is not a trivial task, though, and requires a lot of sophisticated inference derived from a thorough knowledge of the current functional contexts. The discovery of “Lucy’s” footprints preserved in the fossil ashes of a volcano (Leakey and Hay 1979) allowed to conclude on the origin of human bipedal locomotion, but only because this type of movement was already well known from detailed studies of extant human movement. Two contributions to the current issue illustrate the art of tracking footprints by reading otherwise curious details of plastid structure and function as consequence of endosymbiotic evolution.

The contribution by Kuroiwa et al. (2020) is using the organisation of the plastid genomes to infer on early eukaryotic evolution. Based on several decades of expertise in algal cell biology, the authors were able to define three classes of plastid genomes. The original, the so-called CN-type, showed one nucleoid in the chloroplast centre, while the SN-type, prevailing in green algae and land plants, exhibits DNA spread in many copies randomly all over the plastid. The third so-called CL-type harbours circular DNA in the plastid periphery and prevails in red and brown algae. The authors work on a primitive red algal model, *Cyanidioschyzon merolae*, which still displays the ancestral CN-type of plastid DNA. In this model organism, the plastid is still tightly coupled to the cell cycle of the “host cell”. For instance, during mitosis, the plastid nucleoid is stretched towards the spindle poles (Imoto et al. 2010). How this ancestral CN-type was later converted into the characteristic CL-type dominating in the red algae and their derivatives, the brown algae, has remained enigmatic. In the current work, they report that, surprisingly, the CL-type can be produced from the original CN-type by simple drying, meaning that the CL-type nucleoid has arisen by de-condensation of the ancestral CN-type. This transition is accompanied by a characteristic beads-on-a-string structure, which is also a peculiar feature of the CL-type nucleoids in brown algae. Thus, the red algal endosymbiont of brown algae seems to have been an organism very similar to the extant *C. merolae*.

The contribution by Ohashi et al. (2020) asks the question: What happens with the protein filamentous temperature-sensitive Z (FtsZ) during development of the male gametophyte in higher plants? FtsZ executes cell division in prokaryotes and is still needed for the division of the plastid (although the gene is not any longer encoded on the plastid genome but was transferred to the nuclear genome of the “host”, such that the protein has to be imported back to the plastid). In the germinating pollen tube (which is an evolutionary remnant of an own, haploid, generation), the protein is found in the vegetative cell, while it is absent from the germ line (as are the plastids themselves). This might mean that the FtsZ protein is not translated even though mRNA accumulates in the male generative cell or that the transcript is exported into the vegetative cell, where it is translated, or that the transcripts are translated only after fertilisation, such that the FtsZ protein is acting in zygote or the endosperm. Using a GFP fusion of FtsZ in the model plant *Arabidopsis thaliana* (Fujiwara et al. 2010), they show that FtsZ protein is indeed hardly detectable in the germ line, while it is present in the vegetative cell. Furthermore, there is no evidence for a model, where the transcript is transmitted to zygote or endosperm and the protein functions after fertilisation. The finding that a factor crucial for plastid division is specifically suppressed in the male germ line provides a mechanism for the phenomenon that plastidic genes are exclusively inherited through the mother in most plants. Whether it is the absence of a plastid in the germ line interferes with the translation of the transcript, or whether a gradient of the protein in the pollen cell is responsible for the asymmetric partitioning of plastids to germ line and soma, is a fascinating question for future research.

Both contributions allow to infer quite far-reaching conclusions based on seemingly simple, but highly specific observations. Both contributions also reflect what is needed for successful tracking footprints: very clear and specific questions. These questions do not emerge by themselves from a flood of “unbiased”, high-throughput data but have to be mined with a lot of effort based on long experience and thorough knowledge of a cellular phenomenon, and very clear questions.
